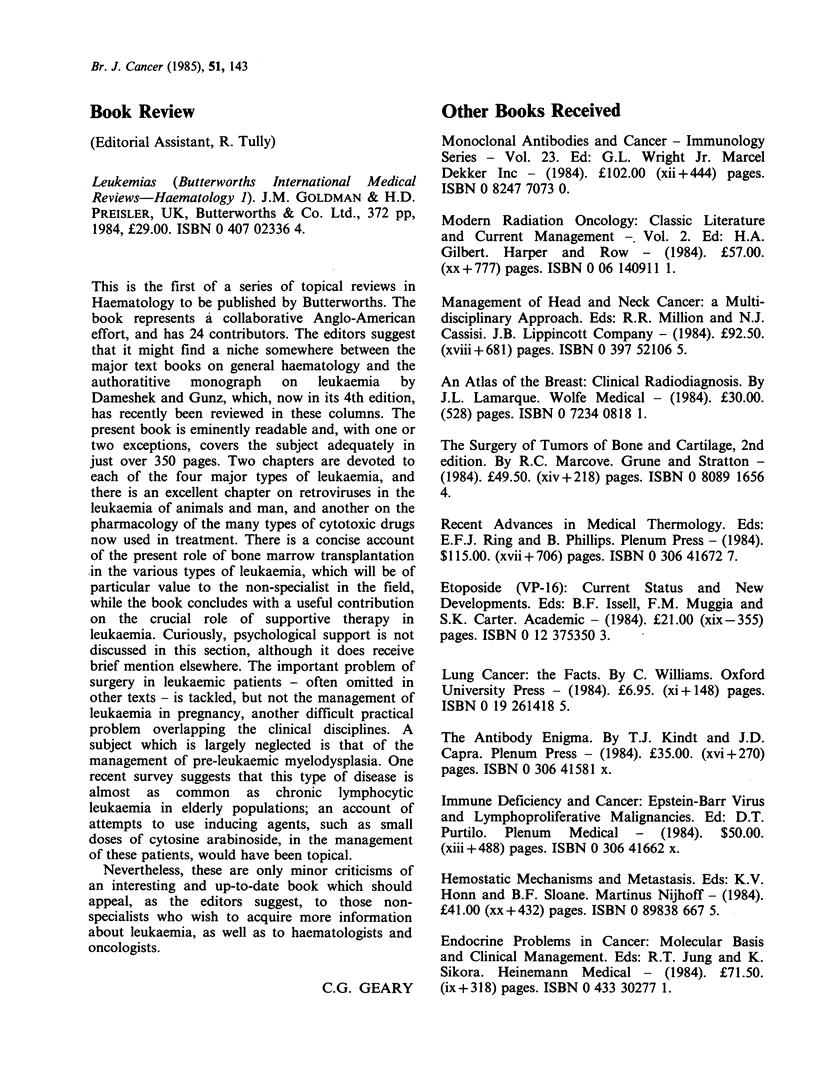# Leukemias (Butterworths International Medical Reviews—Haematology 1)

**Published:** 1985-01

**Authors:** C.G. Geary


### Book Review

(Editorial Assistant, R. Tully)

*Leukemias* (*Butterworths International Medical Reviews—Haematology 1*). J.M. GOLDMAN & H.D. PREISLER, UK, Butterworths & Co. Ltd., 372 pp, 1984, £29.00. ISBN 0 407 02336 4.

This is the first of a series of topical reviews in Haematology to be published by Butterworths. The book represents à collaborative Anglo-American effort, and has 24 contributors. The editors suggest that it might find a niche somewhere between the major text books on general haematology and the authoratitive monograph on leukaemia by Dameshek and Gunz, which, now in its 4th edition, has recently been reviewed in these columns. The present book is eminently readable and, with one or two exceptions, covers the subject adequately in just over 350 pages. Two chapters are devoted to each of the four major types of leukaemia, and there is an excellent chapter on retroviruses in the leukaemia of animals and man, and another on the pharmacology of the many types of cytotoxic drugs now used in treatment. There is a concise account of the present role of bone marrow transplantation in the various types of leukaemia, which will be of particular value to the non-specialist in the field, while the book concludes with a useful contribution on the crucial role of supportive therapy in leukaemia. Curiously, psychological support is not discussed in this section, although it does receive brief mention elsewhere. The important problem of surgery in leukaemic patients – often omitted in other texts – is tackled, but not the management of leukaemia in pregnancy, another difficult practical problem overlapping the clinical disciplines. A subject which is largely neglected is that of the management of pre-leukaemic myelodysplasia. One recent survey suggests that this type of disease is almost as common as chronic lymphocytic leukaemia in elderly populations; an account of attempts to use inducing agents, such as small doses of cytosine arabinoside, in the management of these patients, would have been topical.

Nevertheless, these are only minor criticisms of an interesting and up-to-date book which should appeal, as the editors suggest, to those non-specialists who wish to acquire more information about leukaemia, as well as to haematologists and oncologists.

C.G. GEARY